# Purchasing online journal access for a hospital medical library: how to identify value in commercially available products

**DOI:** 10.1186/1742-5581-3-8

**Published:** 2006-07-12

**Authors:** Thomas P Carter, Anne O Carter, Gwendolyn Broomes

**Affiliations:** 1 School of Clinical Medicine and Research, University of the West Indies, Cave Hill Campus, Barbados; 2 Librarian, Queen Elizabeth Hospital, St. Michael, Barbados

## Abstract

**Background:**

Medical practice today requires evaluating large amounts of information which should be available at all times. This information is found most easily in a digital form. Some information has already been evaluated for validity (evidence based medicine sources) and some is in unevaluated form (paper and online journals). In order to improve access to digital information, the School of Clinical Medicine and Research at the University of the West Indies and Queen Elizabeth Hospital decided to enhance the library by offering online full text medical articles and evidence based medicine sources. The aim of this paper is to evaluate the relative value of online journal commercial products available for a small hospital and medical school library.

**Methods:**

Three reference standards were chosen to represent the ideal list of core periodicals for a broad range of medical care: 2 Brandon/Hill selected lists of journals for the small medical library (BH and BH core) and the academic medical library core journal collection chosen for the Florida State University College of Medicine Medical Library. Six commercially available collections were compared to the reference standards and to the current paper journal subscription list as regards to number of journals matched and cost per journal matched. Ease of use and presence of secondary sources were also considered.

**Results:**

The cost per journal matched ranged from US $ 3194 to $ 81. Because of their low subscription prices, the Biomedical Reference Collection and Proquest products were the most cost beneficial. However, they provided low coverage of the ideal lists (12 – 17% and 21–32% respectively) and contained significant embargoes on current editions, were not user friendly and contained no secondary sources. The Ovid Brandon/Hill Plus Collection overcame these difficulties but had a much higher cost-benefit range while providing higher coverage of the ideal lists (14–47%).

**Conclusion:**

After considering costs, benefits, ease of use, embargoes, presence of secondary sources (ACP Journal Club, DARE), the Ovid Brandon/Hill Plus Collection was the best choice for our hospital considering our budget. However, the option to individually select our own journal list from Ovid and pay per journal has a certain appeal as well.

## Background

Evidence based practice is the conscientious, explicit and judicious use of current best evidence in making decisions about the health care of individuals and groups. The process of evidence based practice involves creating a clinical question from a problem arising from the care of a patient, searching the literature for evidence to answer the question, appraising the evidence found and applying the evidence to the clinical problem [1]. If evidence based practice is to be introduced into routine medical care in an acute care hospital, health care providers and students need immediate access to evidence 24 hours per day, 7 days per week. The traditional concept of a hospital medical library housing a limited collection of scholarly journals with a librarian who can send requests for interlibrary loan of articles not available locally is no longer sufficient. When a health care provider has a clinical question and performs an online search to find the evidence for the answer, the evidence may be found in any of the thousands of scholarly journals currently produced in the biomedical literature although certain "core" journals are more likely to be cited. And the information may well be needed at a time when the librarian is not available. In addition, the wait for an interlibrary loan may well exceed the time period in which health care decisions must be made. The solution to this problem is found in obtaining access to the full text of scholarly publications online.

Many subscriptions to collections of online scholarly publications are available. All are quite costly. Obtaining access to the thousands of biomedical journals currently published is not financially practical for a hospital library with a limited budget. Decision-makers must identify a collection of journals that are most likely to be cited that can be purchased within the library budget. They could benefit from a tool to assist with decision-making as to which of these online collections represents the best value for cost.

The Queen Elizabeth Hospital and the University of the West Indies School of Clinical Medicine and Research, Cave Hill Campus decided to implement increased awareness and use of evidence based practice techniques in the care of patients. It was decided that online access to scholarly journals and other sources of evidence was essential to this process. The two organizations share a building near Bridgetown, Barbados. They also share a library service that, at the time the decision was made to provide online access to journals, was providing traditional service of a limited number of subscriptions to journals stored on site and an interlibrary loan service. The hospital is a 600 bed facility with a budget of approximately US $ 70,000 for scholarly journal subscriptions. The UWI Cave Hill medical campus provides instruction for the final 2 years of undergraduate physician training and supports all phases of postgraduate training in most of the common specialties. The medical school had no specific budget for scholarly journals or other sources of evidence but was willing to start contributing to online information sources.

## Methods

Three reference standards were chosen to represent the "ideal" list of core publications for a broad range of medical care: 1.) the widely recognized Brandon/Hill selected list of journals for the small medical library (BH), 2.) the BH special category of 60 journals considered the minimal core list [2] (BH Core) and 3.) the academic medical library core journal collection chosen for the Florida State University College of Medicine Medical Library [3,4] (FSU). The former two are slanted to the needs of a community hospital and the latter to the needs of a medical school. FSU was chosen because it is a totally new medical school that is very oriented towards evidence based medicine. The lists focus primarily on journals published in the United States but contain journals from many other countries as well. It was also decided that, in addition to scholarly journals (primary sources), a number of sources of summaries of evidence (secondary sources) would be needed. It was decided not to purchase any online textbooks at this time. Commercial providers of online scholarly journals were identified and contacted. Each was asked to provide the list or lists of the journals they could provide and the costs of those lists. Each commercial list was compared to the two BH lists, the FSU list and the list of journals currently subscribed to by the hospital library for completeness. The number of journals on each commercial list that was also on each of the "ideal" core lists was compared with the price of the commercial list. Availability of secondary sources of evidence and ease of use was also considered.

## Results and discussion

Six journal packages (A [5], B [6], C [7], D [7], E [7], F [7]) provided by commercial services were chosen as possible providers of the online scholarly journals for evaluation. They are summarized in Table 1 and are valid only for the date they were submitted for evaluation (October 2003) as these lists change frequently. Only Ovid Technologies could provide sources of summaries of evidence (Cochrane Database of Systematic Reviews, Database of Abstracts of Reviews of Effectiveness and ACP Journal Club) along with the full text of scholarly journals. This would cost US $ 2093 in addition to any journal list chosen. Only Ovid technologies provided software to allow the searcher to go immediately to a citation from the results of a search. With the other providers, a search would need to be conducted elsewhere and articles retrieved by entering the site of the provider.

**Table 1:** Characteristics of Products Evaluated

	**Name of List**	**Name of Provider available**	**Number of journal editions full text**	**Embargo on recent**	**Cost US $**
A	Biomedical Reference Collection Comprehensive Edition	EBSCO Publishing	550	Yes up to 12 months	8215
B	Proquest Research Library, Pharmaceutical News Index and Medical Library	Proquest	494	Yes up to 12 months	7500
C	Ovid Core Biomedical Collection	Ovid Technologies Inc	15	No	12,437
D	Ovid Lippincott Williams & Wilkins Brandon Hill Collection	Ovid Technologies Inc	20	No	11,069
E	Ovid Brandon/Hill Plus Collection	Ovid Technologies Inc	66	No	54,746
F	Ovid Brandon/Hill plus Hague Collection 1 and 11	Ovid Technologies Inc	124	No	99,000

The BH list contains 143 journals but 2 were eliminated as they were indexes of other journals leaving 141 journals (Appendix 1) (Table 2 #2). The BH core list contains 60 journals (Table 2 #1).

**Table 2:** Number & Price per Journal in the Reference Standards for Products Evaluated

**Name of List (number of full text journals on list)**	**#1 Number (%) & price per journal of BH core 60 journals present on list (US $)**	**#2 Number (%) & price per journal of BH 141 journals present on list (US $)**	**#3 Number (%) and price per journal of FSU 449 journals present on list (US $)**	**#4 Number (%) and price per journal of Queen Elizabeth Hospital 208 Journal collection (US $)**
A Biomedical Reference Collection Comprehensive Edition (550)	7 (12%) $1174	17 (12%) $483	75 (17%) $110	23 (11%) $357
B Proquest Research Library, Pharmaceutical News Index and Medical Library (494)	19 (32%) $395	44 (31%) $170	93 (21%) $81	46 (22%) $163
C Ovid Core Biomedical Collection (15)	9 (15%) $1382	13 (9%) $957	15 (3%) $829	8 (4%) $1384
D Ovid Lippincott Williams and Wilkins Brandon Hill Collection (20)	10 (17%) $1107	18 (13%) $615	18 (4%) $615	8 (4%) $1384
E Ovid Brandon/Hill Plus Collection (66)	28 (47%) $1955	64 (45%) $855	64 (14%) $855	27 (13%) $2028
F Ovid Brandon/Hill plus Hague Collection l and ll (124)	31 (52%) $3194	67 (48%) $1478	110 (25%) $900	47 (23%) $2106

The FSU list contains 449 journals (Table 2 #3). The BH list was almost completely subsumed in the FSU list with 136 of the 141 being on it and all of the BH Core being on it. The Queen Elizabeth Hospital library subscribed to 208 paper journals in 2003 (Table 2 #4).

The results of comparison of each journal list evaluated with the reference standards are presented in Table 2. The price per journal ranged from US $ 3194 for F (Ovid Brandon/Hill plus Hague I and II for journals on the #1 BH minimal core list) to a low of US $ 81 per journal for B (Proquest for journals on the #3 FSU list). The US $ 3194 per journal for F covered 52% of the #1 BH minimum core list. In contrast, the cost for E (Ovid BH Plus collection for journals on the #1 BH minimal core list) was US $ 1955 per journal and covered 47% of same collection.

Because of their low subscription prices, the Biomedical Reference Collection and Proquest lists were the most cost- beneficial per frequently used journal. However, they fell far short of the "ideal list". In addition, they contained significant embargoes on current editions and did not contain any secondary sources. Finally, they would not be as user friendly as the Ovid lists since there was no direct link between search result screens and the full text journal article. Despite the higher cost to benefit of the Ovid lists, the lack of embargoes, the availability of secondary sources and user friendly design made them more attractive. Ovid also would allow a library to select individual online journals rather than choosing a fixed list. Any online journals available from Ovid could be selected by our medical library and the cost would be the sum of the individual journals chosen.

Within the Ovid collections, the Ovid Brandon/Hill Plus collection and Ovid Brandon/Hill plus Hague Collection I and II provided high coverage of the "ideal" lists but were very expensive.

## Conclusion

When considering the costs, benefits, ease of use, embargoes, presence of secondary sources, it was decided that the Ovid Brandon/Hill Plus Collection was the best choice for the Queen Elizabeth Hospital/University of the West Indies School of Clinical Medicine and Research Library facility. This fits within the budget available if paper journal subscriptions were to be cancelled. A second option would be to pick individual journals available from Ovid after surveying users.

An additional major financial consideration beyond the cost of the subscriptions considered here was the necessity for more computers and a higher bandwidth network for the library if paper subscriptions were to be cancelled.

The decision as to which commercial collection to purchase will be different for any other medical facility as needs and budgets differ and the contents and prices of the lists available change over time. However, the methods described here to arrive at a decision can be utilized by any facility.

## Competing interests

The author(s) declare that they have no competing interests.

## Authors' contributions

All authors contributed equally to collecting data, analyzing the findings and writing the final product.
